# Oro-facial-digital syndrome type I: a case report with novel features

**DOI:** 10.4322/acr.2021.315

**Published:** 2021-08-20

**Authors:** Shaheen Syed, Poonam Ramnath Sawant, Anita Spadigam, Anita Dhupar

**Affiliations:** 1 Goa Dental College & Hospital, Department of Oral and Maxillofacial Pathology, Bambolim, Goa, India

**Keywords:** Mutation, Hamartoma, Cleft Palate, Ciliopathies

## Abstract

Oro-facial-digital syndrome is a group of rare heterogeneous hereditary disorders characterized by abnormalities of the oral cavity, face and digits, along with varying degrees of mental retardation. Currently, Oro-facial-digital syndrome has been classified into 14 types and two additional unclassified variants have been proposed. Amongst the various variants described, Oro-facial-digital syndrome type I is the most common. We report an interesting subclinical sporadic case of Oro-facial-digital syndrome type I in a 21-year-old female patient. Interestingly, our patient presented with a few novel hitherto unreported clinical findings like midline pits in the philtrum area and a hamartomatous proliferation of tissue in the anterior maxillary alveolar gingival region. This case report highlights the importance of prudent histopathological-clinical correlation, which can direct the flow of clinical investigations leading to the detection and diagnosis of unsuspected conditions as learned in this case. We would also like to emphasize that comprehensive examination of new born for structural abnormalities of the orofacial region is crucial to early diagnosis of syndromes and subsequent referral for further evaluation and management.

## INTRODUCTION

Oro-facial-digital syndrome (OFDS) is a group of rare heterogeneous hereditary disorders characterized by morphogenetic impairment of the oral cavity, face and digits, along with varying degrees of mental retardation, almost limited to the female gender. Currently, OFDS have been classified into 14 types and two additional unclassified variants have been proposed. Amongst the various variants described, OFDS type I is the most commonly presented syndrome and yet is quite rare.^1^^,^ 2

The first description of OFDS syndrome was given by Mohr in 1941, where he reported a family with significant abnormalities of the oral cavity, face and digits. Oro-facial-digital syndrome type I was first reported in 1954, by Papillon-League and Psaume, hence it is also known as Papillon-League-Psaume syndrome. In 1964, Gorlin & Pindborg coined the term ‘Orodigitofacial dysostosis’. However, due to reports of multi-organ involvement the term ‘Oro-facial-digital syndrome’ is preferred.^3^

OFDS I is inherited as an X-linked dominant condition, which is lethal to with variable degree of expression within the same family. The gene responsible for this disorder is found on the short arm of the X chromosome (Xp22.3-p22.2). In a study by Ferrante et al.^4^ mutations in the *CXORF5* gene were detected, which was later termed *OFD1* gene (MIM# 311200).^4^

It has been reported that, approximately, 75% of the OFDS I cases are sporadic. Sometimes a female proband with OFDS I may have the disorder as a result of de novo pathogenic variant.^5^^,^ 6 The incidence of OFDS I is 1:50000 to 1:250000 live births and the prevalence is estimated to be between 1 out of 25,000 to 1 out of 150,000 live births.^5^^,^ 6 ^,^ 7

Syndromes show variable expressivity, necessitating recognition and differential diagnosis of the clinical presenting signs and symptoms. A case of oro-facial-digital syndrome type I, with special clinical aspects is presented, highlighting the importance of interpreting histopathological features in the detection and unmasking of unsuspected conditions including syndromes.

## CASE REPORT

An excisional biopsy of an anterior maxillary gingival growth was received for routine histopathological examination from a 21-year-old female patient presenting for treatment of mal-aligned anterior teeth. The provisional diagnosis was a fibroma.

**Figure 1:**
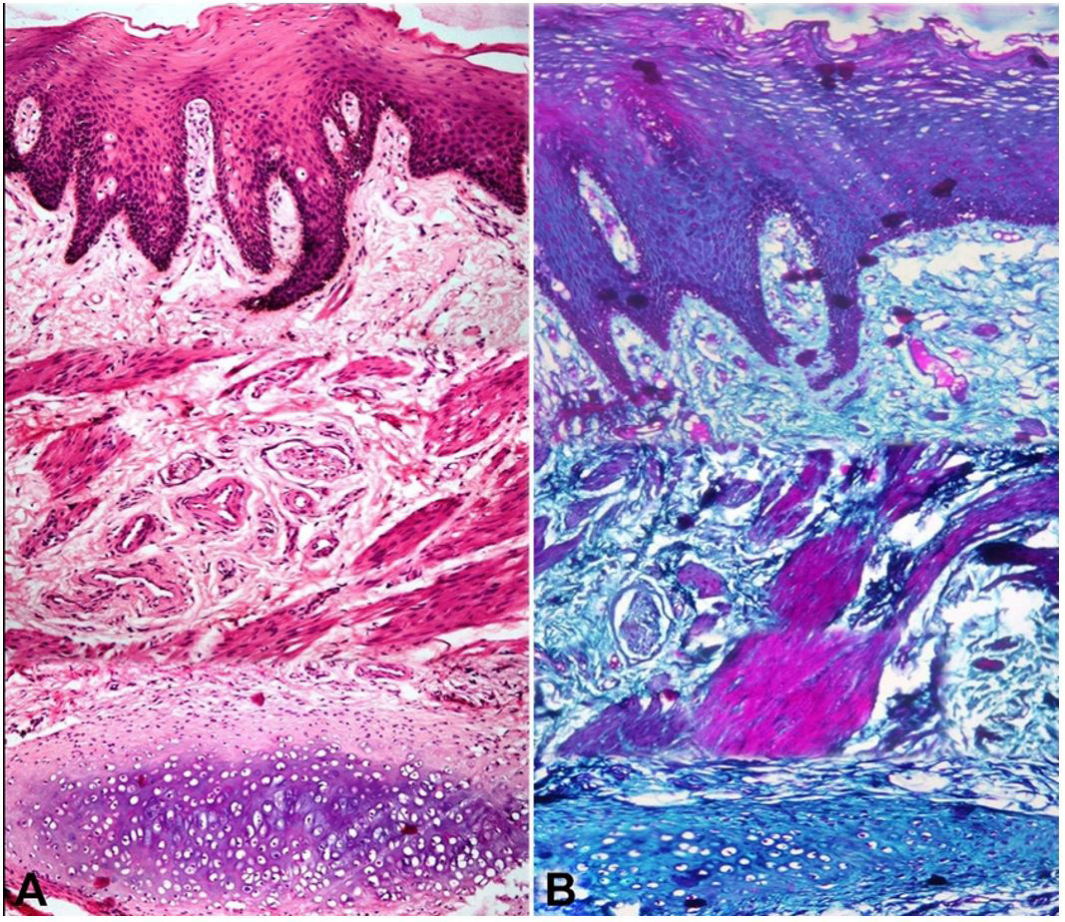
Photomicrographs of the biopsied soft tissue lesion: **A** – stratified squamous para-keratinized epithelium overlying a fibro-cellular connective tissue stroma predominantly comprising of blood vessels, neural tissue, smooth muscle bundles and forming hyaline cartilage in deeper stroma (H&E, 10X); **B** – Masson trichrome stain used to differentiate the smooth muscle cells (stained pink) from dense collagen fibres (stained blue) (10X).

The histopathological evaluation of the Hematoxylin and eosin-stained sections of the biopsied tissue showed stratified squamous parakeratinizing epithelium overlying a fibro cellular stroma. The stroma consisted of loosely arranged collagen fibers, loosely arranged bundles of differentiated smooth muscle fibers, nerve fascicles, thick-walled blood vessels and ectopic cartilaginous tissue (Figure 1A). The Masson Trichrome special stain was used to delineate the different connective tissue components (Figure 1B).

As the histopathological findings were suggestive of a hamartoma, a comprehensive clinical anamnesis with radiographic investigations was requisitioned.

**Figure 2:**
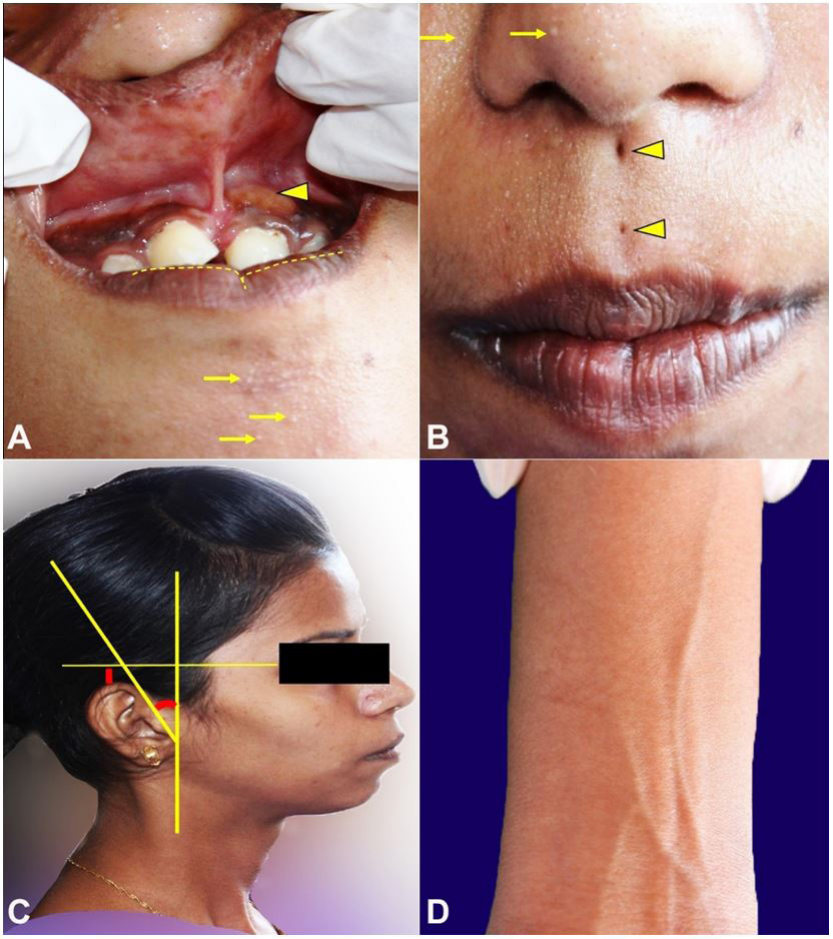
Clinical examination: **A** – showing soft tissue swelling over the alveolar mucosa (arrow head), abnormal frenal attachment, midline diastema, median alveolar cleft, mesio-labial rotation of the right central incisor, pseudo cleft of the lower lip (yellow dotted line), milia (arrows); **B** – Philtrum pits (arrowhead), milia (arrows); **C** – Low set ears; **D** – Thin scanty hair.

Clinical examination revealed a normal-statured, well-oriented female in apparent good health. Extra-orally, micrognathia, pseudo-clefting of the lower lip, two midline pits in the labial philtrum and low set ears were evident (Figure 2A-C). The skin of patient was dry with thin scanty hair, crops of milia were noted on the nose, along the nasolabial folds and chin (Figure 2A, B, D).

Intra-oral examination of the patient revealed mesio-labial rotation of the right maxillary central incisor, a midline diastema associated with a median alveolar cleft, high labial frenal attachment and an additional small soft tissue gingival swelling (measuring approximately 1x1.5 cm in dimension) in relation to the left maxillary central incisor (Figure 2A) .

There was no evidence of malformation of hands and feet and her medical history was unremarkable. Given this constellation of signs, the patient’s mother was interviewed. The mother confirmed that she had a non-consanguineous marriage, the patient was delivered as a premature baby with low birth weight (exact weight not known) and had learning difficulties. She also mentioned that the patient has a completely normal younger male sibling.

**Figure 3:**
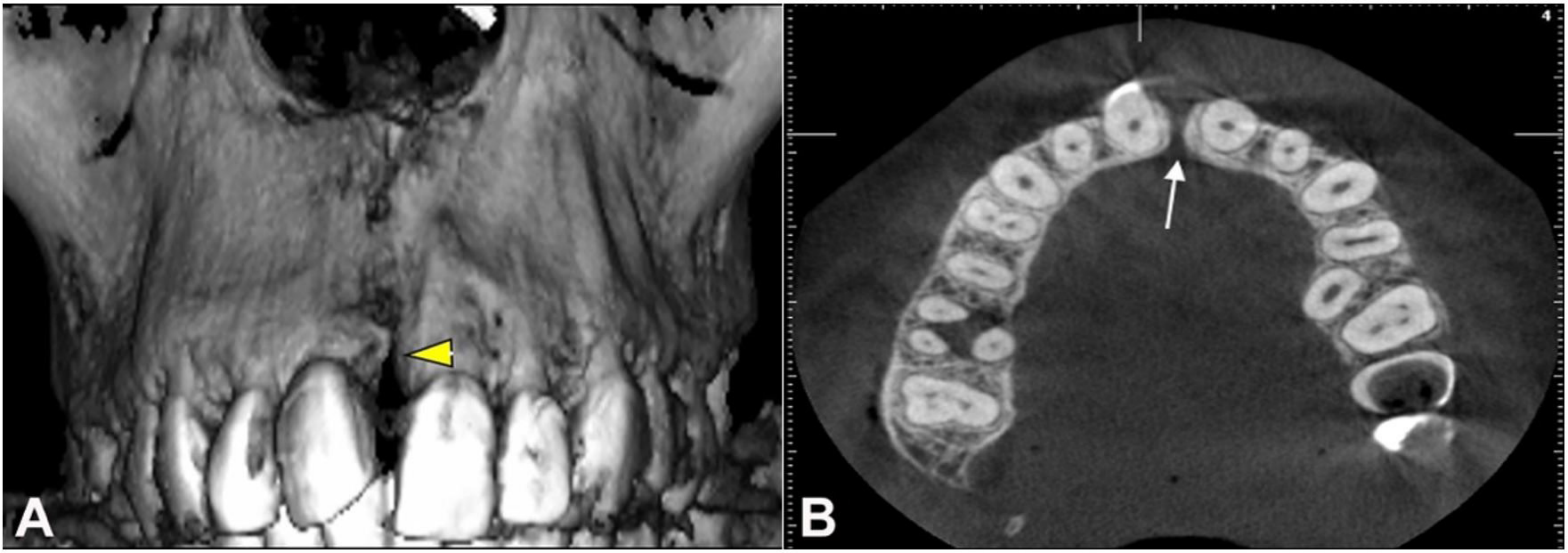
Tomographic examination of maxilla showing median alveolar cleft. **A** – 3D reconstruction; **B** – Axial view.

The patient was advised an orthopantamograph (OPG), lateral cephalogram, cone beam computed tomography (CBCT) of the jaw bones and an abdominal ultrasound to rule out polycystic kidney disease. The CBCT (Figure 3A, B) and the OPG confirmed the presence of a median alveolar cleft of the maxilla, while the abdominal ultrasound was unremarkable.

A karyotyping test was conducted. The test revealed an apparently normal karyotype as assessed by conventional cytogenetic analysis (CCA). A review of literature suggested that a normal karyotype has been reported in patients with clinical diagnosis of Oro-facial-digital syndrome type I, as not all genetic mutations are identifiable by CCA and requires use of advanced molecular genetic testing methods to ascertain the clinical diagnosis. In the present case a clinical diagnosis of Oral-facial-digital syndrome type I was concluded upon based on the clinic-radiographic features. In this case advanced molecular genetic tests were not conducted due to financial reasons and thus remains to be a limitation of this case report.

Clinical management of such cases is multidisciplinary and depends on the severity of phenotypic expression of the mutated OFD I gene. Since our patient was not aware of her medical condition and did not present with major anatomical defects, she was informed and counseled about the same and advised to keep in touch for a regular follow up.

## DISCUSSION

Oro-facial-digital syndrome type I is a rare, X-linked dominant male lethal ciliopathy with variable clinical presentation owing to varied mutations within the *OFDI* gene (*CXORF5*). The OFD I protein is localized to the basal body of the primary cilia.^8^

**Figure 4:**
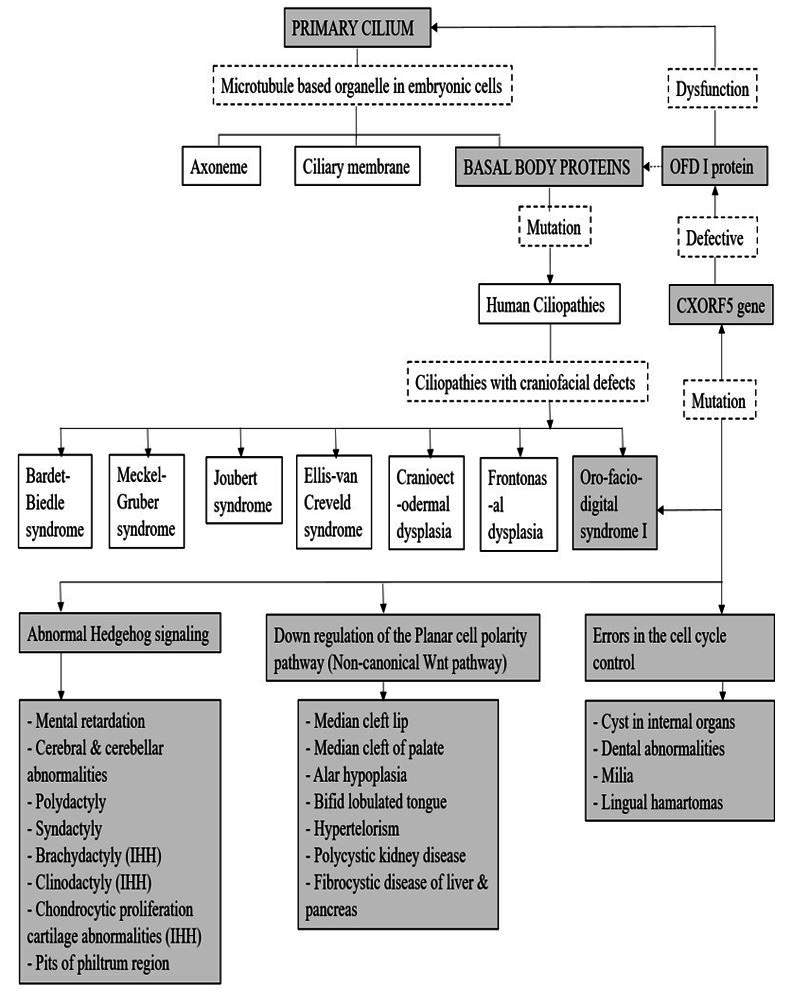
Pathogenesis of Oro-facial-digital syndrome type I, according to AlKattan et al., 2015^11^.

The term ‘primary cilium’ was coined by Sergei Sorokin, to describe an organelle that emanates from the cell surface of most mammalian cell types during growth arrest. The primary cilium provides a means of sequestering the centriole, thus majority of the cells that have primary cilia are non-cycling differentiated cells or stem cells in G0 phase.^9^^,^ 10 The primary cilia are found on different cell types in the human body, including the stem, epithelial, endothelial, muscle, connective tissue and the neuronal cells. Increasing evidence suggests that the primary cilium is the key coordinator during development and in tissue homeostasis. Primary cilia also plays a vital role in modulating cell signaling pathways. Experimental studies have shown that, various receptors, ion channels, transporter proteins, downstream effector molecules, are localized to the basal body. Thus, primary cilium helps orchestrate key developmental processes like cell migration, cell differentiation, cell cycle control, plane of cell division and apoptosis. The signaling pathways modulated at the level of the basal body of the primary cilium are diverse and depend on the cell type.^9^ Genetic mutations in any of the proteins associated with the basal body of the primary cilium can result in various human diseases or syndromes, which are collectively known as ‘Human ciliopathies’. The OFDI protein is one of the proteins associated with the basal body of the primary cilium, when defective results in the clinical manifestations of the OFD I syndrome. The molecular pathogenesis of OFDS type I has been presented in a simplified format using a flow chart (Figure 4).^11^

Through this case report, we aim to highlight a subclinical sporadic case of OFDS type I, which lacked the easily observable phenotypic features of the syndrome and presented with few novel hitherto unreported clinical findings. To the best of our knowledge, we report the first patient of OFDS type I with midline pits in the philtrum area and a hamartomatous proliferation of tissue in the anterior maxillary alveolar gingival region showing exuberant proliferation of smooth muscle cells, blood vessels, neural tissue and cartilaginous tissue.

The planar cell polarity (PCP) pathway is known to orchestrate proper orientation, migration and intercalation of the tissue cells and the Indian hedgehog pathway (IHH) is associated with chondrocyte proliferation. Thus, as described in Figure 3, down regulation of the PCP pathway and abnormal functioning of the IHH pathway coupled with abnormal cycle control, may have led to the philtrum pits and hamartoma formation in our patient.^11^ The cartilaginous tissue could have arisen from abnormal proliferation of the remnants of embryonic cartilage precursors from nasal and septal development in the anterior part of the maxilla.^12^

While our patient had a limited expression of the conventional phenotypic features, she presented with philtrum pits and hamartomatous proliferation of soft tissues of the anterior maxillary gingiva, thus representing yet another facet in the varying phenotypic spectrum of OFDS type I.

In order to ease the clinical evaluation and diagnosis of the varied spectrum of Oro-facial-digital syndromes and the syndromes showing features overlapping with OFDS type I, the authors performed a thorough review of literature and tabulated their clinical features (Table 1) and genetic aberrations (Table 2) for a quick easy review.

**Table 1:** Comparative analysis of the clinical features evident in Oro-facial-digital syndrome type I and other syndromes constituting its differential diagnosis (EVC = Ellis-van Creveld syndrome; JS = Joubert syndrome; MGS = Meckel-Gruber syndrome; PHS = Pallister-Hall syndrome; SLOS = Smith-Lemli-Opitz syndrome)

Clinical features/ differential diagnosis			I	II	III	IV	V	VI	VII	VIII	IX	X	XI	XII	XIII	XIV	UI	U II	EVC	JS	MGS	PHS	SLOS
			Types of OFDS																				
Extra oral features																							
Stature [1,5] (Short +, Normal -)			+	-	-	+	-	-	-	-	+	+	-	-	-	-	-	-	+	-	-	-	+
Eye	Hypertelorism^1^		+	+	+	-	-	+	+	+	+	-	+	+	-	-	-	-	-	+	+	-	-
	Blepharophimosis^1^		-	-	-	-	-	-	-	-	-	-	+	-	-	-	-	-	-	-	-	-	-
	Coloboma^1^		-	-	-	-	-	-	-	-	+	-	-	-	-	-	-	-	-	+	+	-	-
	Exophthalmos^2^		-	-	-	-	-	-	-	-	-	+	-	-	-	-	-	-	-	-	-	-	-
	Seesaw winking^2^^,^ 5		-	-	+	-	-	-	-	-	-	+	-	-	-	-	-	-	-	-	-	-	-
	Epicanthus fold^1^		-	-	-	+	-	-	-	-	-	-	-	-	-	-	-	-	-	-	-	-	-
	Telecanthus^1^		-	-	-	-	-	-	-	+	-	-	-	-	-	+	-	-	-	-	-	-	-
	Synophrys, Microphthalmia^1^^,^ 5		-	-	-	-	-	-	-	-	+	-	-	-	-	-	-	-	-	-	+	-	-
	Retinal abnormalities^1^^,^ 5		-	-	-	-	-	-	-	-	+	-	-	-	-	-	-	-	-	-	-	-	-
	Epicanthus fold^1^		-	-	-	-	-	-	-	-	-	-	-	-	-	-	-	-	-	-	+	-	+
	Ptosis^13^		-	-	-	-	-	-	-	-	-	-	-	-	-	-	-	-	-	+	-	-	+
	Nystagmus^13^		-	-	-	-	-	-	-	-	-	-	-	-	-	-	-	-	-	+	-	-	-
	Oculomotor apraxia^13^		-	-	-	-	-	-	-	-	-	-	-	-	-	-	-	-	-	+	-	-	-
	Hypoplastic optic nerve^14^		-	-	-	-	-	-	-	-	-	-	-	-	-	-	-	-	-	-	+	-	-
	Strabismus^13^		-	-	-	-	-	-	-	-	-	-	-	-	-	-	-	-	+	+	-	-	-
	Congenital cataracts^15^		-	-	-	-	-	-	-	-	-	-	-	-	-	-	-	-	+	-	-	-	+
	Blepharosis^16^		-	-	-	-	-	-	-	-	-	-	-	-	-	-	-	-	-	-	-	-	+
	Microphthalmia^14^		-	-	-	-	-	-	-	-	-	-	-	-	-	-	-	-	-	-	+	-	-
Nose	Broad bifid tip^17^		-	+	-	-	-	-	-	-	-	-	-	-	-	-	-	-	-	-	-	-	-
	Broad nasal root^17^		-	+	-	-	-	-	-	+	-	-	-	-	-	-	-	-	-	-	-	-	-
	Bulbous nose^1^^,^ 5		-	-	+	+	-	-	-	+	-	-	-	-	-	-	-	-	-	-	+	+	-
	Flat nasal root^1^		-	-	-	-	-	-	-	-	-	+	-	-	-	-	-	-	-	-	-	-	-
	Hypoplasia of the alae^11^		+	-	-	-	-	-	-	-	-	-	-	-	-	-	-	-	-	-	-	-	-
	Hypoplastic nasal septum^14^		-	-	-	-	-	-	-	-	-	-	-	-	-	-	-	-	-	-	+	-	-
	Short nose upturned nostrils^18^		-	-	-	-	-	-	-	-	-	-	-	-	-	-	-	-	-	-	-	+	+
	Broad or flat nasal bridge^18^		-	-	-	-	-	-	-	-	-	-	-	-	-	-	-	-	-	-	-	+	-
	Nostrils turned forward^16^		-	-	-	-	-	-	-	-	-	-	-	-	-	-	-	-	-	-	-	-	+
Ear	Hearing defects^17^		+	+	-	-	-	-	-	-	-	-	-	-	-	-	-	-	-	-	-	-	-
	Low set ears^1^^,^ 5		+	-	+	+	-	-	-	-	-	-	-	-	-	-	-	-	-	-	+	-	-
	Abnormal inner ear^1^		-	-	-	-	-	-	-	-	+	-	-	-	-	-	-	-	-	-	-	-	-
	Auricular pits & Deafness^1^		-	-	-	-	-	-	-	-	-	-	+	-	-	-	-	-	-	-	-	-	-
	Malformed ear^14^		-	-	-	-	-	-	-	-	-	-	-	-	-	-	-	-	-	-	+	-	-
	Small ears rotated backwards^18^		-	-	-	-	-	-	-	-	-	-	-	-	-	-	-	-	-	-	-	+	-
	Large external ears^14^^,^ 16		-	-	-	-	-	-	-	-	-	-	*-*	-	-	-	-	-	-	-	+	-	+
Intra oral region:																							
Palate	Cleft palate^1^^,^ 17		+	+	+	+	-	+	+	+	+	+	+	-	+	+	+	-	-	+	+	-	+
Lip	Median cleft lip^17^		+	+	+	+	+	+	-	+	+	-	-	-	-	-	+	-	-	-	-	+	+
	Cleft lip^1^		-	-	-	-	-	+	+	-	-	-	-	-	+	-	-	-	-	+	+	-	-
	Short upper lip^15^		-	-	-	-	-	-	-	-	-	-	-	-	-	-	-	-	+	-	-	-	-
	Midline long vertical groove in upper lip^18^		-	-	-	-	-	-	-	-	-	-	-	-	-	-	-	-	-	-	-	+	-
	Long inverted V shape upper lip^16^		-	-	-	-	-	-	-	-	-	-	-	-	-	-	-	-	-	-	-	-	+
Tongue	Cleft^1^		-	+	-	-	-	-	-	-	-	-	-	-	-	+	-	-	-	-	-	-	-
	Lobulated tongue^1^		+	+	+	+	-	+	-	+	+	-	-	-	-	+	+	-	-	+	+	+	-
	Bifid or Trifid^1^		+	-	-	-	-	-	-	-	-	-	-	+	-	-	-	-	-	+	-	-	-
	Lingual hamartomas^1^		+	+	+	+	-	+	+	+	+	+	-	-	+	+	-	+	-	-	+	+	-
	Ankyloglossia^19^		+	-	-	-	-	-	-	-	-	-	-	-	-	-	-	-	-	-	-	-	-
	Bifid uvula^1^		-	-	+	-	-	-	-	-	-	-	-	-	-	-	-	-	-	-	+	-	-
	Cleft epiglottis^14^		-	-	-	-	-	-	-	-	-	-	-	-	-	-	-	-	-	-	+	-	-
	Microglossia^18^		-	-	-	-	-	-	-	-	-	-	-	-	-	-	-	-	-	-	-	+	-
	Cleft or fissure in the larynx^18^		-	-	-	-	-	-	-	-	-	-	-	-	-	-	-	-	-	-	-	+	-
	Epiglottis hypoplasia^1^^,^ 5		-	-	-	-	-	-	-	+	-	-	-	-	-	-	-	-	-	-	-	-	-
	Bifid epiglottis^18^		-	-	-	-	-	-	-	-	-	-	-	-	-	-	-	-	-	-	-	+	-
	Inflexible epiglottis^1^^,^ 2		-	-	-	-	-	-	-	-	+	-	-	-	-	-	-	-	-	-	-	-	-
Gingiva	Gingival Frenulae^1^		+	+	-	+	+	+	+	+	+	+	+	+	-	+	+	-	+	+	-	+	-
	Labiogingival adherence^15^		-	-	-	-	-	-	-	-	-	-	-	-	-	-	-	-	+	-	-	-	-
	Submucosal clefts^15^		-	-	-	-	-	-	-	-	-	-	-	-	-	-	-	-	+	-	-	-	-
	Labial vestibule obliteration^15^		-	-	-	-	-	-	-	-	-	-	-	-	-	-	-	-	+	-	-	-	-
	Buccal frenula^18^		-	-	-	-	-	-	-	-	-	-	-	-	-	-	-	-	-	-	-	+	-
	Abnormal gums^16^		-	-	-	-	-	-	-	-	-	-	-	-	-	-	-	-	-	-	-	-	+
Dentition	Missing teeth^17^		+	+	-	-	-	-	-	-	-	-	-	-	-	-	-	-	-	-	-	-	-
	Supernumerary teeth^1^		+	-	+	-	-	-	-	-	-	-	-	+	-	-	-	-	-	-	+	-	-
	Diastema^15^		-	-	-	-	-	-	-	-	-	-	-	-	-	-	-	-	+	-	-	-	-
	Conical teeth^15^		-	-	-	-	-	-	-	-	-	-	-	-	-	-	-	-	+	-	-	-	-
	Natal teeth^18^		-	-	-	-	-	-	-	-	-	-	-	-	-	-	-	-	-	-	-	+	-
	Neonatal teeth^14^^,^ 15		-	-	-	-	-	-	-	-	-	-	-	-	-	-	-	-	+	-	+	-	-
	Hypodontia^15^		-	-	-	-	-	-	-	-	-	-	-	-	-	-	-	-	+	-	-	-	-
	Enamel dysplasia^11^		+	-	-	-	-	-	-	-	-	-	-	-	-	-	-	-	-	-	-	-	-
	Enamel hypoplasia^1^		-	-	+	-	-	-	-	-	-	-	-	-	-	-	-	-	+	-	+	-	-
	Tooth malformations^2^		-	-	-	-	-	-	-	-	-	+	-	-	-	-	-	-	-	-	-	-	-
	Premature eruption^15^		-	-	-	-	-	-	-	-	-	-	-	-	-	-	-	-	+	-	-	-	-
	Premature exfoliation^15^		-	-	-	-	-	-	-	-	-	-	-	-	-	-	-	-	+	-	-	-	-
Mandible	Hypoplastic mandible^17^		+	+	-	-	-	-	-	-	-	-	-	-	-	-	-	-	+	-	-	-	-
	Micrognathia^14^		+	-	-	+	-	-	-	-	-	-	-	-	-	-	-	-	+	-	+	-	-
	Retrognathia^1^		-	-	-	-	-	-	-	-	-	+	-	-	-	-	-	-	-	-	+	-	-
	Short mandible^16^		-	-	-	-	-	-	-	-	-	-	-	-	-	-	-	-	-	-	-	-	+
	Jaw winking^2^				+	-	-	-	-	-	-	-	-	-	-	-	-	-	-	-	-	-	-
Digits [Hands &Feet]	Brachydactyly^1^		+	+	-	+	-	+	-	-	+	-	-	-	+	-	-	-	-	-	-	-	-
	Clinodactyly^1^		+	+	-	+	-	+	+	-	+	-	-	-	+	-	-	-	-	-	+	-	-
	Polydactyly^1^		+	+	+	+	+	+	-	+	+	+	+	+	-	+	+	+	+	+	+	+	+
	Syndactyly^1^		+	-	-	-	-	+	-	-	-	-	-	-	+	-	-	-	-	-	+	+	+
	Oligodactyly^1^		-	-	-	-	-	-	-	-	-	+	-	-	-	-	-	-	-	-	-	-	-
Others systems																							
Skin		Milia^5^	+	-	-	-	-	-	-	-	-	-	-	-	-	-	-	-	-	-	-	-	-
Hair		Thick hair^1^	-	+	-	-	-	-	-	-	-	-	-	-	-	-	+	-	-	-	-	-	-
		Thin dry hair^11^	+	-	-	-	-	-	-	-	-	-	-	-	-	-	-	-	+	-	-	-	-
		Alopecia^11^	+	-	-	-	-	-	-	-	-	-	-	-	-	-	-	-		-	-	-	-
		Photosensitivity^16^	-	-	-	-	-	-	-	-	-	-	-	-	-	-	-	-	-	-	-	-	+
CNS		Mental retardation^1^^,^ 17	+	+	+	+	+	+	-	+	-	-	-	+	-	+	+	-	-	+	-	-	+
		Epilepsy^2^^,^ 5 ^,^ 16 ^,^ 18	-	-	+	-	-	-	-	-	-	-	-	-	+	-	-	-	-	-	-	+	+
		Intellectual disability^1^^,^ 5	-	-	+	-	-	-	+	+	-	-	+	-	-	+	+	-	-	-	-	-	-
		Psychomotor retardation^2^	-	-	-	-	-	-	-	-	-	+	-	-	-	-	-	-	-	-	-	-	-
		Macrocephaly^1^	-	-	-	-	-	-	-	-	-	-	-	+	-	-	-	-	-	-	-	-	-
		Microcephaly^14^^,^ 16 ^,^ 18	-	-	-	-	-	-	-	-	-	-	-	-	-	-	-	-	-	-	+	+	+
		Neuropsychiatric troubles^1^	-	-	-	-	-	-	-	-	-	-	-	-	+	-	-	-	-	-	-	-	-
		Encephalocele^13^^,^ 14	-	-	-	-	-	-	-	-	-	-	-	-	-	-	-	-	-	+	+	-	-
		Hydrocephaly^14^^,^ 16 ^,^ 18	-	-	-	-	-	-	-	-	-	-	-	-	-	-	-	-	-	-	+	+	+
		Anencephaly^14^^,^ 16 ^,^ 18	-	-	-	-	-	-	-	-	-	-	-	-	-	-	-	-	-	-	+	+	+
		Cerebellar vermis agenesis^14^	-	-	-	-	-	-	-	-	-	-	-	-	-	-	-	-	-	-	+	-	+
		Malformed hypothalamus^18^	-	-	-	-	-	-	-	-	-	-	-	-	-	-	-	-	-	-	-	-	-
CVS		Coarctation of the aorta^1^	-	-	-	-	-	-	-	-	-	-	-	-	-	-	-	+	-	-	-	-	-
		Single atrium^15^	-	-	-	-	-	-	-	-	-	-	-	-	-	-	-	-	+	-	-	-	-
		Defects of the mitral and tricuspid valves^15^	-	-	-	-	-	-	-	-	-	-	-	-	-	-	-	-	+	-	-	-	-
		Patent ductus arteriosus^15^	-	-	-	-	-	-	-	-	-	-	-	-	-	-	-	-	+	-	+	-	-
		Septum hypertrophy^1^	-	+	-	-	-	-	-	-	-	-	-	+	-	-	-	-	-	-	-	-	-
		Valve dysplasia^1^	-	-	-	-	-	-	-	-	-	-	-	-	+	-	-	-	-	-	-	-	-
		Ventricular septal defect^1^	-	-	-	-	-	-	-	-	-	-	-	-	-	-	+	-	+	-	+	-	-
		Atrial septal defect^14^^,^ 15	-	-	-	-	-	-	-	-	-	-	-	-	-	-	-	-	+	-	+	-	-
		Hypoplastic left heart syndrome^15^	-	-	-	-	-	-	-	-	-	-	-	-	-	-	-	-	+	-	-	-	-
		Congenital heart defects^16^^,^ 18	-	-	-	-	-	-	-	-	-	-	-	-	-	-	-	-	-	-	-	+	+
Kidney		Kidney absent^5^^,^ 13	+	-	+	+	-	+	-	-	-	-	-	-	-	-	-	-	-	-	-	-	-
		Polycystic kidney disease^1^	+	-	+	+	-	-	+	+	-	-	-	-	-	-	-	-	-	+	+	+	-
		Renal dysplasia^1^^,^ 5	-	-	-	-	-	+	-	-	-	-	-	-	-	-	-	-	-	+	+	-	-
		Renal failure^1^	-	-	+	-	-	-	-	-	-	-	-	-	-	-	-	-	-	+	-	-	-
		Fused kidneys^1^	-	-	-	-	-	-	-	-	-	-	-	-	-	-	+	-	-	-	-	-	-
		Agenesis of kidney^18^	-	-	-	-	-	-	-	-	-	-	-	-	-	-	-	-	-	-	-	+	-
Liver		Macrocysts^5^	+	-	-	-	-	-	-	-	-	-	-	-	-	-	-	-	-	-	-	-	-
		Fibrosis^2^^,^ 14	-	-	-	-	-	-	-	-	-	-	-	-	-	-	-	-	-	-	+	-	-
Pancreas		Macrocysts^5^	+	-	-	-	-	-	-	-	-	-	-	-	-	-	-	-	-	-	-	-	-
Ovary		Macrocysts^5^	+	-	-	-	-	-	-	-	-	-	-	-	-	-	-	-	-	-	-	-	-
Skeletal system		Y-shaped metacarpal^1^	-	+	-	-	-	+	-	-	-	-	-	+	-	-	-	+	-	-	-	-	-
		Tibia abnormalities^1^	-	-	-	+	-	-	-	+	-	-	-	+	-	-	-	-	-	-	-	-	-
		Radius hypoplasia^1^	-	-	-	-	-	-	-	+	-	+	-	-	-	-	-	-	-	-	-	-	-
		Fibular agenesis^1^	-	-	-	-	-	-	-	-	-	+	-	-	-	-	-	-	-	-	-	-	-
		Vertebral abnormalities^1^	-	-	-	-	-	-	-	-	-	-	+	-	-	-	-	-	-	-	-	-	-
		Shortening of the middle and distal phalanges^15^	-	-	-	-	-	-	-	-	-	-	-	-	-	-	-	-	+	-	-	-	-
		Deformity of the knees or lumbar lordosis^15^	-	-	-	-	-	-	-	-	-	-	-	-	-	-	-	-	+	-	-	-	-
		Bowing of the long bones of limbs^14^	-	-	-	-	-	-	-	-	-	-	-	-	-	-	-	-	-	-	+	-	-
		Talipes equinovarus^14^	-	-	-	-	-	-	-	-	-	-	-	-	-	-	-	-	-	-	-	-	-
		Abnormally short arms and/or legs and/or dislocated hips^18^	-	-	-	-	-	-	-	-	-	-	-	-	-	-	-	-	-	-	-	+	-

**Table 2:** Genotypic variation seen in Oro-facial-digital syndrome type I and other syndromes constituting its differential diagnosis (EVC = Ellis-van Creveld syndrome; JS = Joubert syndrome; MGS = Meckel-Gruber syndrome; PHS = Pallister-Hall syndrome; SLOS = Smith-Lemli-Opitz syndrome)

Type	Phenotype MIM# Number	Inheritance Pattern	Gene	Cytogenetic location
Type I^20^	311200	X linked dominant	*CXORF5*	Xp22.3-p22.2
Type II^20^	-	Autosomal recessive	*Unidentified gene*	-
Type III^20^	258850	Autosomal recessive	*TMEM231*	16q23.1
Type IV^20^	258860	Autosomal recessive	*TCTN3*	10q24.1
Type V^20^	174300	Autosomal recessive	*DDX59*	1q32.1
Type VI^20^	277170	Autosomal recessive	*C5ORF42*	5p13.2
Type VII^20^	608518	X-linked dominant	*-*	-
Type VIII^20^	300484	X-linked recessive	*-*	-
Type IX^20^	258865	Autosomal recessive	*TBC1D32*	6q22.31
Type X^20^	-	Sporadic	*-*	-
Type XI^20^	-	Sporadic	*-*	-
Type XII^20^	-	Sporadic	*-*	-
Type XIII^20^	-	Sporadic	*-*	-
Type XIV^20^	615948	Autosomal recessive	*C2CD3*	11q13.4
Unclassified OFD^20^	613580	Autosomal recessive	*WDPCP*	2p15
Unclassified OFD^20^	617563	Autosomal recessive	*TMEM107*	17p13.1
EVC^15^	225500	Autosomal recessive	*EVC and EVC2*	4p16.2
JS 10^13^	300804	Autosomal recessive	*OFD1*	Xp22.2
MGS^14^	614209	Autosomal recessive	*B9D1*	17p11.2
	614175		*B9D2*	19q13.2
	612284		*CC2D2A*	4p15.32
	611134		*CEP290*	12q21.32
	249000		*MKS1*	17q22
	611561		*RPGRIP1L*	16q12.2
	613885		*TCTN2*	12q24.31
	258860		*TCTN3*	10q24.1
	607361		*TMEM67*	8q22.1
	617562		*TMEM107*	17p13.1
	603194		*TMEM216*	11q12.2
	615397		*TMEM231*	16q23.1
	614424		*TMEM237*	2q33.1
PHS^18^	607324	Autosomal Dominant	*GLI3*	7p13.1
SLOS^16^	270400	Autosomal recessive	*DHCR7*	11q13.4

## CONCLUSION

We contribute to the existing literature, previously unreported features of OFDS I and propose the inclusion of philtrum pits and hamartoma involving any of the oral mucosal tissue (not limited to the tongue) in the clinical presentation of OFDS type I.

Through this case the authors would like to highlight the significance of noting unusual histopathological findings in routine specimens with reappraisal of clinical data which may be crucial to diagnosis of such cases, which are rare and have shown subclinical presentation. The patients diagnosed with OFDS type I are at a risk of developing polycystic kidney disease, hence need to be kept under observation with careful morphological assessment and biochemical monitoring. We would also like to emphasize that comprehensive examination of new born for structural abnormalities of the orofacial region is crucial to early diagnosis and subsequent referral for further evaluation.
